# One-Step Preparation of High-Purity Sodium Tungstate from Wolframite via Alkali Fusion and the Mechanism of Impurity Directional Migration

**DOI:** 10.3390/ma19050932

**Published:** 2026-02-28

**Authors:** Hailong Bai, Liwen Zhang, Xiaoli Xi, Zuoren Nie

**Affiliations:** 1 State Key Laboratory of Materials Low-Carbon Recycling, Beijing University of Technology, Beijing 100124, China; baihailong_2024@163.com (H.B.); zhangliwen@bjut.edu.cn (L.Z.); zrnie@bjut.edu.cn (Z.N.); 2 Collaborative Innovation Center of Capital Resource-Recycling Material Technology, College of Materials Science and Engineering, Beijing University of Technology, Beijing 100124, China

**Keywords:** wolframite, sodium tungstate, alkali fusion, phase separation, slag regulation

## Abstract

The extraction of high-purity sodium tungstate from complex wolframite concentrates presents significant challenges due to the limitations of conventional processing methods, which are often energy-intensive and generate substantial secondary waste. In this study, we propose a novel phase-regulated alkali fusion approach for the one-step production of high-purity Na_2_WO_4_. Using phase-diagram calculations with FactSage in the Na-Fe-Mn-Si-O system, SiO_2_ was introduced to regulate slag formation, promoting immiscibility between the silicate slag and Na_2_WO_4_ melt. This resulted in a clear stratification of the phases at 1000 °C, enabling spontaneous separation of the Na_2_WO_4_-rich salt phase from the slag. The optimized conditions achieved a sodium tungstate purity of 98.76%, with a tungsten recovery rate of 98.91%. Furthermore, impurity elements such as Fe and Mn were preferentially retained in stable silicate/oxide phases within the slag, contributing to the high purity of the sodium tungstate product. This method offers a simplified and environmentally friendly alternative to traditional hydrometallurgical and pyrometallurgical processes, with significant implications for the efficient utilization of complex tungsten resources.

## 1. Introduction

Tungsten (W), as a strategic metal, is indispensable in defense and aerospace industries due to its extremely high melting point and hardness [1,2]. Currently, economically viable tungsten resources are primarily derived from wolframite ((Fe,Mn)WO_4_) and scheelite (CaWO_4_) [3]. With the depletion of easily processable high-grade ores, tungsten resources are trending toward complex compositions and low grades [4,5]. Wolframite is typically beneficiated (e.g., via gravity and magnetic separation) to produce a concentrate before further extraction. Wolframite commonly occurs with multiple impurity minerals (e.g., quartz, sulfide ores), and its inherent iron and manganese components combined with complex mineralogical characteristics pose significant challenges for traditional processing methods, including low extraction efficiency and difficulties in impurity separation [6,7]. Therefore, developing efficient and environmentally friendly extraction technologies specifically targeting complex wolframite ores is crucial for ensuring sustainable supply of tungsten resources.

Currently, the industrial processing methods for wolframite primarily rely on acid leaching [8]. Although thermodynamically feasible, this process is significantly constrained kinetically: the acid leaching forms a dense tungstic acid (H_2_WO_4_) passivation film on the mineral surface, which severely hinders mass transfer and results in low decomposition rates [9,10,11]. To enhance leaching efficiency, mechanical activation, ultrafine grinding, or pressure leaching [12] are often required. These intensification measures not only substantially increase process complexity and energy consumption but may also introduce new pollution. In contrast, the high-pressure alkaline leaching method suitable for wolframite [13,14,15,16] faces challenges including high alkali consumption, high-salinity wastewater generation, and near-complete dissolution of molybdenum as Na_2_MoO_4_ during processing. The subsequent molybdenum removal using Na_2_S further complicates the process flow, making it economically unfavorable for wolframite treatment. Therefore, whether employing acidic or alkaline approaches, existing hydrometallurgical routes all exhibit inherent limitations in terms of efficiency, cost, or environmental friendliness when processing complex wolframite.

To fundamentally address the wastewater and residue issues generated by hydrometallurgy, pyrometallurgical routes have regained attention. However, early pyrometallurgical studies (e.g., using fluxing agents such as Na_2_CO_3_ and Na_2_SiO_3_) primarily focused on scheelite processing and were typically conducted at high temperatures (>1100 °C), often resulting in low-value-added intermediate products [17,18,19]. Although Kim’s study based on the Na_2_O-WO_3_-SiO_2_ ternary phase diagram demonstrated the feasibility of achieving phase separation through controlled slag composition [20], these investigations have primarily employed relatively simple compositional wolframite systems as model frameworks. There remains a lack of systematic exploration into complex and variable wolframite mineral systems, particularly regarding insufficient understanding of migration patterns for major impurity elements such as Fe and Mn generated during wolframite decomposition processes.

Based on previous studies by scholars, it can be observed that there exists a research gap in the current literature: specifically, the lack of a solution specifically targeting the characteristics of wolframite to achieve efficient decomposition, clean phase separation, and impurity regulation. The high energy consumption and low-quality products associated with traditional pyrometallurgical methods originate from the absence of effective control over the slag–salt phase separation process and the migration behavior of impurity elements.

Therefore, this study proposes a novel low-temperature alkali fusion synergistic phase control method specifically targeting wolframite. During the initial research phase, it was found that simple alkali fusion could not achieve separation between sodium tungstate products and the slag phase. Through thermodynamic equilibrium calculations using FactSage software (8.2 version), it was determined that regulating SiO_2_ additive composition enables phase separation. Consequently, SiO_2_ was introduced into the NaOH melting system as a fluxing agent, with the dual purpose of not only promoting decomposition but more critically, inducing spontaneous separation of wolframite decomposition products at 1000 °C into a high-purity sodium tungstate liquid phase and a silicate slag phase capable of effectively capturing impurities through modulation of slag phase properties. After cooling, the slag and salt naturally stratify, enabling physical separation of slag and salt phases. The produced sodium tungstate meets enterprise purity standards. The innovation of this study lies in:

1. A novel low-temperature alkali fusion synergistic phase control method specifically targeting wolframite was proposed.
2. The directional migration and fixation mechanisms of impurity elements were elucidated.
3. The discharge of high-salinity wastewater inherent in conventional processes was avoided.

## 2. Experimental Methods

### 2.1. Materials

**Figure 1:**
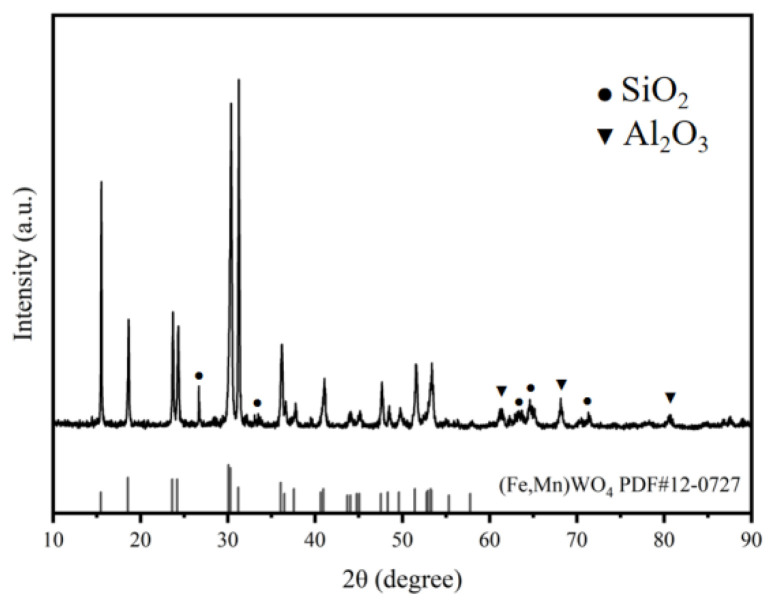
XRD of wolframite.

After grinding the wolframite (supplied by China Nonferrous Metals (Guangxi) Pinggui Feidie Co., Ltd., Hezhou China), X-ray diffraction (XRD) analysis was conducted. The XRD results shown in Figure 1 indicate that the wolframite is primarily composed of (Fe,Mn)WO_4_. According to the XRF results presented in Table 1, the content of wolframite accounts for about 83.5 wt.% of the total mass of the sample, among which the proportions of iron wolframite (FeWO_4_) and manganese wolframite (MnWO_4_) are 61.1 wt.% and 22.4 wt.%, respectively, corresponding to an Fe/Mn mass ratio of approximately 2.7:1, indicating that the ore belongs to iron-rich wolframite. The main associated minerals include quartz (SiO_2_, approximately 4.5 wt.%), galena (PbS, approximately 3.4% wt.), and pyrite (FeS_2_, approximately wt.1.5%). The remaining components consist of trace elements such as calcium, aluminum, and potassium silicates. The sum of the X-ray fluorescence (XRF) analysis results was normalized to 100%, demonstrating good data quality. This compositional characteristic indicates that W, Fe, and Mn are primarily hosted within the wolframite, while Pb and part of Fe independently form galena and pyrite, respectively. AR-grade NaOH was purchased from Aladdin Reagent Co., Ltd. (Shanghai, China). AR-grade SiO_2_ was obtained from Fuchen Reagent Co., Ltd. (Tianjin, China).

### 2.2. Experiment Procedure

**Figure 2:**
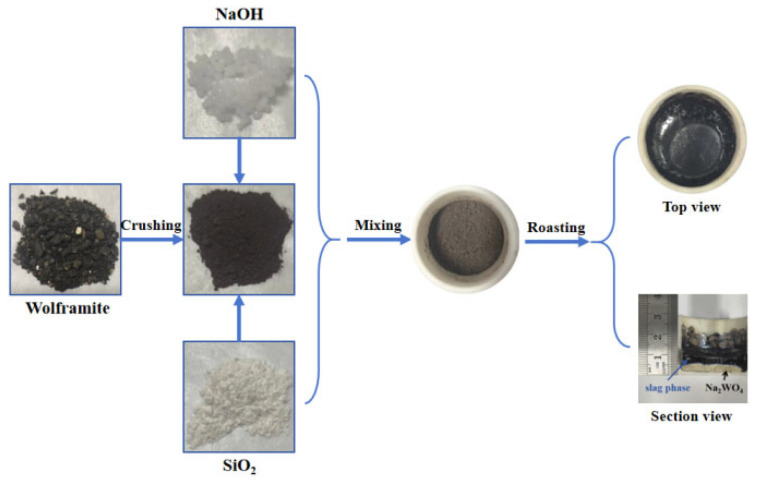
Schematic diagram of alkali fusion phase separation.

This study initially employed sodium hydroxide alkali fusion to decompose wolframite. Under controlled temperature conditions, the tungsten components in wolframite reacted with sodium hydroxide to form water-soluble sodium tungstate and a slag phase. However, these two phases remained mixed together, preventing the separation of pure sodium tungstate product. To achieve effective separation between the soluble sodium tungstate and the slag phase while improving product purity, phase diagram calculations were conducted using FactSage software (8.2 version) to guide subsequent process design. The optimized process, as illustrated in Figure 2, involved adding silica into the alkali fusion system. The introduced silica reacted with impurity components to form a stable silicate slag phase, enabling efficient one-step separation of sodium tungstate from the slag. Experimental results demonstrated that this modified process not only significantly simplified the workflow but also yielded high-purity Na_2_WO_4_ with excellent metallurgical properties and economic benefits.

#### 2.2.1. Conventional Alkali Fusion

The alkali fusion test was conducted in a constant-temperature muffle furnace (Model XSB-8-8-12-1C, Thermcraft, Winston-Salem, NC, USA). A mixture of 10 g ore powder and NaOH at a specific molar ratio was placed into a 25 mL alumina cylindrical crucible and reacted in the muffle furnace under an air atmosphere for a certain period (i.e., the chamber was closed, allowing natural air exchange, and no specific gas was introduced). Wolframite reacts with NaOH to form water-soluble Na_2_WO_4_, while Fe and Mn are converted into iron/manganese oxides that remain in the insoluble residue (slag phase). In this study, by varying the molar ratio of NaOH to wolframite (calculated as WO_3_) and adjusting the alkali fusion temperature while keeping the alkali fusion duration constant, the alkali fusion products were washed with deionized water. The residue phase was dried, ground, and subjected to XRF analysis to determine the WO_3_ content in the residue. Based on Equation (1), the leaching rate was calculated to identify the optimal alkali fusion process.

(1) $$\eta_{0}=\frac{{(m}_{1}\times W_{1})-{(m}_{2}\times W_{2})}{m_{1}\times W_{1}}\times 100\%$$

In the formula, *η*_0_ represents the extraction efficiency of wolframite (%), and m_1_ and m_2_ respectively represent the mass of wolframite and the mass of water-washed slag after each reaction, W_1_ and W_2_ respectively represent the WO_3_ content in wolframite and the WO_3_ content in the water-washed slag after the reaction.

#### 2.2.2. Alkali Fusion with Phase Separation

Under an air atmosphere, wolframite concentrate, NaOH, and SiO_2_ were charged into a 25 mL alumina cylindrical crucible. The feed ratios were varied to investigate slag–salt phase separation for one-step preparation of high-purity sodium tungstate. The crucible was heated to 1000 °C and held for 3 h, followed by furnace cooling to room temperature. The cooling rate is 120 °C/h. After cooling, a distinct stratified structure formed, consisting of a Na_2_WO_4_-rich salt layer and an oxide/silicate slag layer. The crucible was mechanically crushed and the two solidified layers were readily detached along the sharp interface, enabling direct physical separation of the Na_2_WO_4_ product from the slag without additional washing. The purity of the collected Na_2_WO_4_ was analyzed, and the recovery was calculated according to Equation (2).

(2) $$\eta_{1}=\frac{m_{1}\times M_{{\mathrm{WO}}_{3}}}{m_{2}\times W_{1}\times M_{{\mathrm{Na}}_{2}{\mathrm{WO}}_{4}}}\times 100\%$$

In the formula, *η*_1_ represents the separation rate, m_1_ is the mass of Na_2_WO_4_ collected, m_2_ is the mass of added wolframite, W_1_ is the WO_3_ content in the surface wolframite, $M_{{\mathrm{WO}}_{3}}$ is the relative molecular mass of WO_3_, and $M_{{\mathrm{Na}}_{2}{\mathrm{WO}}_{4}}$ is the relative molecular mass of Na_2_WO_4_.

The collected sodium tungstate mass is calculated with Equation (3).

(3) $$M=w\times (m_{1}-m_{2})$$

In the formula, M represents the mass of Na_2_WO_4_ collected, w is the purity of the prepared sodium tungstate, m_1_ is the total mass of the product and the crucible after the reaction, and m_2_ is the total mass of the residue phase and the crucible after washing.

### 2.3. Analysis Methods

X-ray fluorescence (XRF) was used to determine the chemical composition of the wolframite concentrate and alkali-fusion slag. Phase identification of the concentrate, slag, and Na_2_WO_4_ product was performed by X-ray diffraction (XRD, TTR III, Cu Kα, Rigaku, Tokyo, Japan) with a step size of 0.02° and a scanning rate of 2°/min. Concentrations of Fe, Mn, S, and As in solution were measured by ICP-OES (iCAP 7400, Thermo Scientific, Waltham, MA, USA). The morphology and elemental distribution of the separated products were characterized by SEM-EDS (JSM-IT500LV, JEOL, Tokyo, Japan).

## 3. Results and Discussion

### 3.1. Thermodynamic Calculations

This section aims to quantitatively evaluate the possibility, directionality, and phase equilibrium relationships of chemical reactions in the research system from a theoretical level through thermodynamic calculations. Calculations are performed separately for the thermodynamics of alkali fusion reactions and phase diagram thermodynamics.

#### 3.1.1. Thermodynamic Calculations for Wolframite Reactions

**Figure 3:**
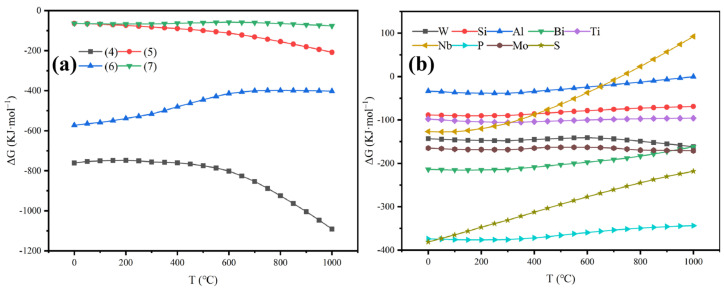
(**a**) ΔG versus temperature for wolframite main reaction. (**b**) ΔG versus temperature for impurity reaction.

In order to study the feasibility of Equations (4)–(7), that is, the feasibility of conversion of Na_2_WO_4_ from FeWO_4_, the thermodynamic parameter (standard Gibbs free energy change ΔG) of the reaction was theoretically calculated. The thermodynamic data of FeWO_4_, MnWO_4_, Na_2_WO_4_, Fe_2_O_3_ and Mn_2_O_3_ are obtained from HSC 6.0. According to the classical thermodynamic theory, the relationship between ΔG and T is calculated, and the results are shown in Figure 3a. Obviously, at 900 °C, the Gibbs free energy of Equations (4)–(7) is less than 0, the reaction can occur spontaneously, the Equation (6) becomes more positive with the increase in temperature, and the Gibbs free energy of Equations (4), (5) and (7) becomes more negative with the increase in temperature. Therefore, Equations (4)–(7) are thermodynamically feasible, and increasing the temperature is beneficial for the thermodynamic conversion of FeWO_4_ and MnWO_4_ to Na_2_WO_4_. The FeWO_4_-NaOH system can achieve tungsten melting under high temperature and oxygen-rich conditions, and controlling the ratio of NaOH to FeWO_4_ greater than 2 is advantageous for achieving a higher degree of conversion from FeWO_4_ toNa_2_WO_4_ during the alkali fusion process.

4FeWO_4_ + 8NaOH + O_2_ → 2Fe_2_O_3_ + 4Na_2_WO_4_ + 4H_2_O (4)

4MnWO_4_ + 8NaOH + O_2_ → 2Mn_2_O_3_ + 4Na_2_WO_4_ + 4H_2_O (5)

FeWO_4_ + 2NaOH → FeO + Na_2_WO_4_ + H_2_O (6)

MnWO_4_ + 2NaOH → MnO + Na_2_WO_4_ + H_2_O (7)

The impurities in wolframite will also react with NaOH to form oxygen-containing acid salts, the reaction equation of which is shown in Equation (8) (M = W, Si, Al, Bi, Ti, Nb, P, Mo, S).

MO_x_ + NaOH → Na_x_M_x_O_x_ + H_2_O (8)

It can be seen from Figure 3b that most impurity elements will participate in the reaction under experimental conditions, and will compete with FeWO_4_ while reacting with NaOH, leading to a decrease in the extraction efficiency of sodium tungstate. Through the calculations in Table 1, it is found that the theoretical molar ratio of WO_3_ and the total of impurity elements to NaOH is 1:2.63. If the extraction efficiency of wolframite is to be improved, the alkali to material molar ratio needs to be greater than 2.6.

#### 3.1.2. Thermodynamic Calculation of Phase Diagram

Previous studies have found that simple alkali fusion, while improving reaction efficiency, results in the mixing of the reaction product Na_2_WO_4_ with the slag phase, making separation impossible. To achieve efficient separation of sodium tungstate from the slag phase in the alkali fusion products of wolframite, this study introduces SiO_2_ as a slag-phase regulator. Under the alkaline conditions of this system (excess NaOH), Na_2_O acts as a network modifier, effectively breaking the Si-O-Si network and forming low-melting-point silicates (e.g., Na_2_SiO_3_). This overall reduction in the viscosity and melting point of the slag phase ensures sufficient fluidity at the experimental temperature to enable gravity separation based on density differences.

**Figure 4:**
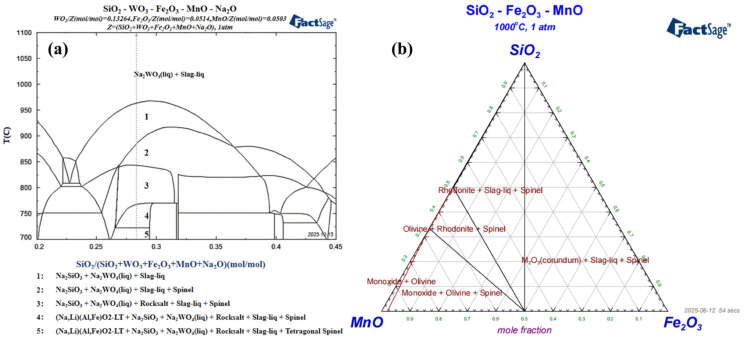
(**a**) SiO_2_-WO_3_-Fe_2_O_3_-MnO-Na_2_O quinary phase diagram. (**b**) SiO_2_-Fe_2_O_3_-MnO ternary phase diagram.

In order to gain an in-depth understanding of the intrinsic mechanism of slag–salt phase separation and guide subsequent work, thermodynamic calculations were performed using FactSage software, and phase diagrams were plotted. After analyzing the composition of wolframite, Fe_2_O_3_, MnO, and WO_3_ were selected as quantitative components. Using the SiO_2_/Z ratio range of 0.2 to 0.45 on the x-axis and temperature as the variable on the y-axis, a phase diagram for the SiO_2_-WO_3_-Fe_2_O_3_-MnO-Na_2_O five-component system was plotted under atmospheric pressure within the temperature range of 700–1100. The results are shown in Figure 4a. When the temperature exceeds 968 °C, a single-phase region appears. Therefore, the subsequent reaction temperature was controlled at 1000 °C. At 1000 °C, only the slag phase and the Na_2_WO_4_ phase exist. In the system at 1000 °C, the Fe_2_O_3_ and MnO phases are miscible with the glassy melt. The Na_2_WO_4_ phase and the slag phase are immiscible, achieving the goal of separation. As can be seen from Figure 4a, when x = 0.285, six phase regions appear between 700 and 1100 °C. In phases 1–5, the Na_2_SiO_3_ phase cannot be removed, ultimately affecting product purity. At 885 °C, the spinel phase and the slag phase dissolve into each other, achieving impurity removal; however, the Na_2_SiO_3_ phase is still not removed. At 1000 °C, the spinel phase, Na_2_SiO_3_ phase, and slag phase become mutually miscible. Only the slag phase and the Na_2_WO_4_ phase remain. Due to their differing densities and immiscibility, the Na_2_WO_4_ phase and the slag phase form distinct layers upon cooling—the slag layer and the sodium tungstate layer—thus achieving separation and obtaining high-purity Na_2_WO_4_. To investigate the migration mechanism of impurities, a phase diagram for the SiO_2_-Fe_2_O_3_-MnO ternary system at 1000 °C was plotted using FactSage software. The results are shown in Figure 4b.

These thermodynamic calculations, based on equilibrium conditions and utilizing the FactPS and FToxid databases, provide critical guidance for achieving phase separation. It should be noted that the constructed multi-component phase diagrams (e.g., Figure 4) simplify the system by focusing primarily on major components (W, Fe, Mn, Si, Na, O), without explicitly incorporating trace impurities (e.g., Pb, S), which are predicted to not significantly alter the dominant slag–salt immiscibility behavior. The validity of this modeling approach for process design is ultimately confirmed. A limitation of the equilibrium model lies in its exclusion of kinetic factors.

### 3.2. Alkali Fusion Process

In order to explore the optimal conditions for tungsten extraction efficiency in the reaction of wolframite with NaOH at atmospheric pressure, a series of conditional experiments were conducted to clarify the effects of alkali dosage molar ratio, alkali fusion temperature, and holding time on extraction efficiency.

#### 3.2.1. Effect of NaOH Stoichiometric Ratio

**Figure 5:**
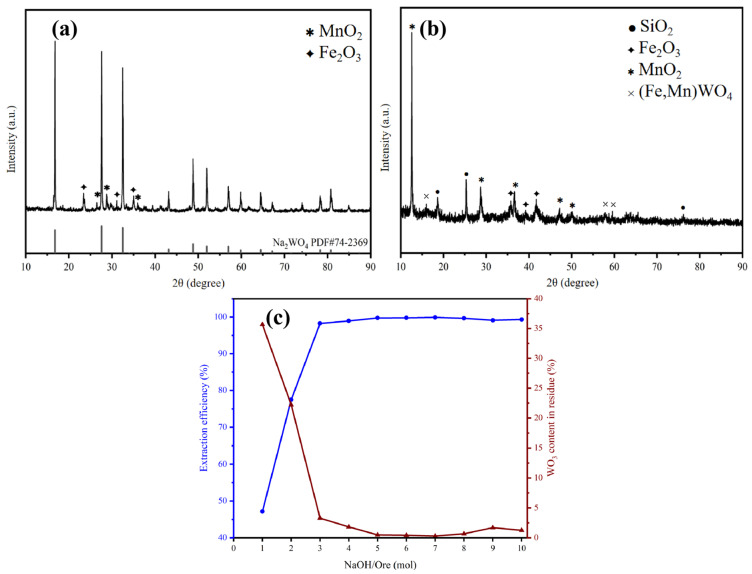
(**a**) XRD pattern of the alkali fusion product at an alkali-to-material ratio of 2:1; (**b**) XRD pattern of the water-washed residue at an alkali-to-material ratio of 2:1; and (**c**) Effect of the molar ratio of alkali to material on extraction efficiency.

The concentration of NaOH is the main driving force in the alkali fusion process, thus it has a significant impact on the melting of tungsten. At 900 °C, with a holding time of 3 h and a NaOH:WO_3_ molar ratio of 2:1, the alkali fusion product is shown in Figure 5a. Under the 2:1 condition, the main phase of the product is Na_2_WO_4_, with a small amount of Fe_2_O_3_ and MnO_2_ present. The XRD results of the water-washed slag are shown in Figure 5b, and some unreacted FeWO_4_, Fe_2_O_3_, and MnO_2_ phases still remain in the slag. By changing the alkali to flux ratio from 1:1 to 10:1 and using Formula (1) to calculate the extraction efficiency, the results are shown in Figure 5c. When the alkali to flux ratio is 4:1, the extraction efficiency can reach 99%, and as the alkali to flux ratio increases, the extraction efficiency remains stable at more than 99%, and the WO_3_ content in the melting product slag drops to 1%. Therefore, the optimal molar ratio for the reaction is determined to be 4:1.

#### 3.2.2. Effect of Alkali Fusion Temperature

**Figure 6:**
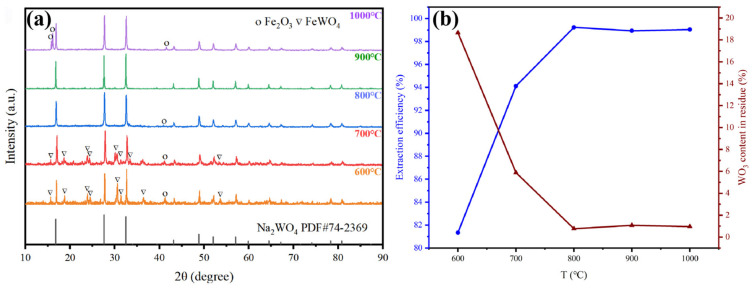
(**a**) XRD pattern of products at 600–1000 °C. (**b**) Effect of alkali fusion temperature on extraction efficiency.

Previous studies have shown that changes in temperature affect the Gibbs free energy, influencing the occurrence of reactions. From the perspective of industrial production, increasing the reaction temperature increases costs. Therefore, determining the optimal reaction temperature is crucial for the industrialization of this method. Under the conditions of an alkali-to-material molar ratio of 4:1 and a holding time of 3 h, the effect of alkali fusion temperature on tungsten extraction efficiency was studied, controlling the temperature between 600 and 1000 °C. The XRD of the products at each temperature is shown in Figure 6a. At 600 and 700 °C, there are still unreacted FeWO_4_ residues in the product. Above 800 °C, the main phase is sodium tungstate, with a small amount of Fe_2_O_3_ impurities present. The impact of temperature on extraction efficiency is shown in Figure 6b. At 800 °C, the extraction efficiency reaches 99.23%, and thus 800 °C is the optimal alkali fusion temperature.

#### 3.2.3. Effect of Holding Time

**Figure 7:**
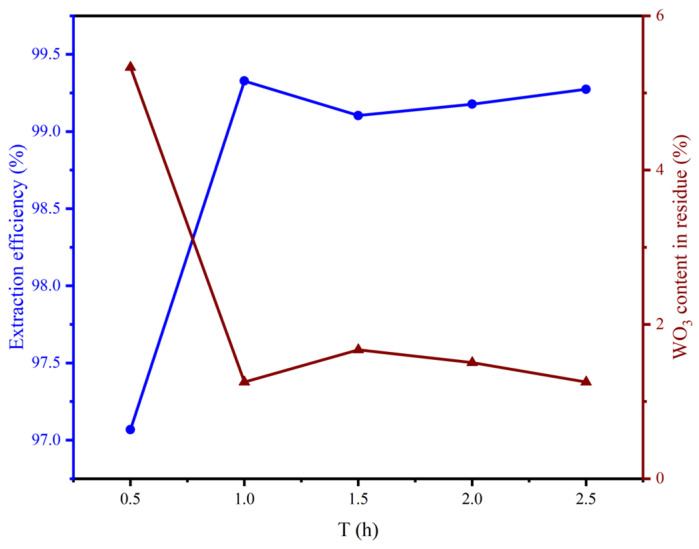
Effect of holding time on the extraction efficiency.

When the alkali fusion process reaches equilibrium, prolonging the reaction time will increase the energy consumption required to maintain the alkali fusion reaction. Therefore, reaction time is also a key factor for industrialization of the technology. Under the conditions of an alkali to material molar ratio of 4:1 and a reaction temperature of 800 °C, the effect of holding time on the tungsten extraction efficiency was investigated, and the results are shown in Figure 7: extraction efficiency of 97% can be achieved under the condition of 0.5 h, and when the holding time is increased to 1 h, the extraction efficiency reaches 99%. Considering the extraction efficiency and production efficiency comprehensively, the most suitable reaction time is determined as 1 h.

### 3.3. Phase Separation of Sodium Tungstate at Moderate Temperature

Section 3.2 determined the optimal conditions (800 °C, 1 h, molar ratio of NaOH to WO_3_ as 4:1) aimed at maximizing the decomposition efficiency of wolframite and the generation of Na_2_WO_4_. However, under these conditions, the generated Na_2_WO_4_-rich phase remains mixed with slag, making direct physical separation impossible; thus, different parameters are required. The selection of 1000 °C is based on thermodynamic phase diagram calculations, which indicate a stable immiscible region between Na_2_WO_4_ melt and silicate slag above approximately 968 °C. Raising the temperature is necessary to ensure effective gravitational separation. Prolonging the holding time to 3 h ensures that the system reaches complete thermodynamic equilibrium, which is critical for forming a clear interface and obtaining high-purity Na_2_WO_4_ products through simple mechanical peeling. Therefore, experiments involving the addition of SiO_2_ to achieve phase separation were conducted at 1000 °C for 3 h.

#### 3.3.1. Slag–Salt Separation Process

**Figure 8:**
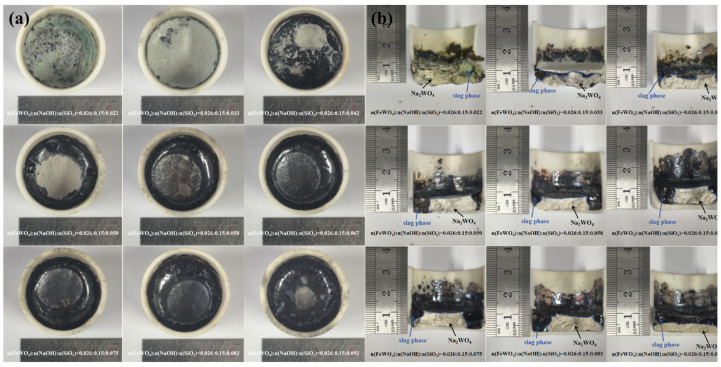
Results of Experiments 1–9 Diagram: (**a**) Top View and (**b**) Sectional View.

To achieve slag–salt phase separation, experiments were conducted in a alumina cylindrical crucible using wolframite, NaOH, and SiO_2_ at 1000 °C for 3 h, while systematically varying the SiO_2_ addition. The specific experimental ratios are shown in Table 2, and the phase separation results are shown in Figure 8. Under the conditions of Experiment 3, stratification began to appear, but sodium tungstate was still entrained in the upper slag phase; under the conditions of Experiment 4, the stratification was clearer, with no obvious entrainment between the slag and salt phases. As the amount of SiO_2_ added increased, the stratification effect became more pronounced, and the glass phase content gradually increased. By the conditions of Experiment 9, the system exhibited a clear two-phase structure from top to bottom, and the thickness of the sodium tungstate layer decreased from the initial 0.5 mm to 0.3 mm.

Insufficient addition of SiO_2_ leads to incomplete phase separation and product entrainment, whereas excessive addition merely increases slag quantity without beneficial effects, thereby directly increasing reagent costs and diluting the potential value of subsequent slag utilization. This creates a narrow operational window requiring precise control, which may necessitate re-optimization for wolframite concentrates with varying compositions (different inherent silicon contents). Considering the separation effect and actual economic benefits comprehensively, the optimal molar ratio of WO_3_:NaOH:SiO_2_ was determined to be 0.0261:0.15:0.05. As shown in Figure 8b, increasing the SiO_2_ dosage changes the slag morphology from encapsulating the salt phase to spreading over it. This transition is associated with enhanced polymerization of silicate species in the slag, which modifies the slag rheology and interfacial properties. As a result, the slag tends to form a continuous thin-film layer and the slag–salt interface becomes clearer, facilitating effective phase separation.

This distinct slag–salt natural stratification is the key to the practicality and environmental friendliness of this process. Compared with the conventional hydrometallurgical route for wolframite that requires multiple steps (such as leaching, solid–liquid separation, purification, etc.) and generates large volumes of high-salinity wastewater, this method simultaneously achieves separation and preliminary purification during the high-temperature process. While simplifying the process flow, it avoids the generation of process wastewater.

#### 3.3.2. Trends in Impurity Elements in Wolframite

**Figure 9:**
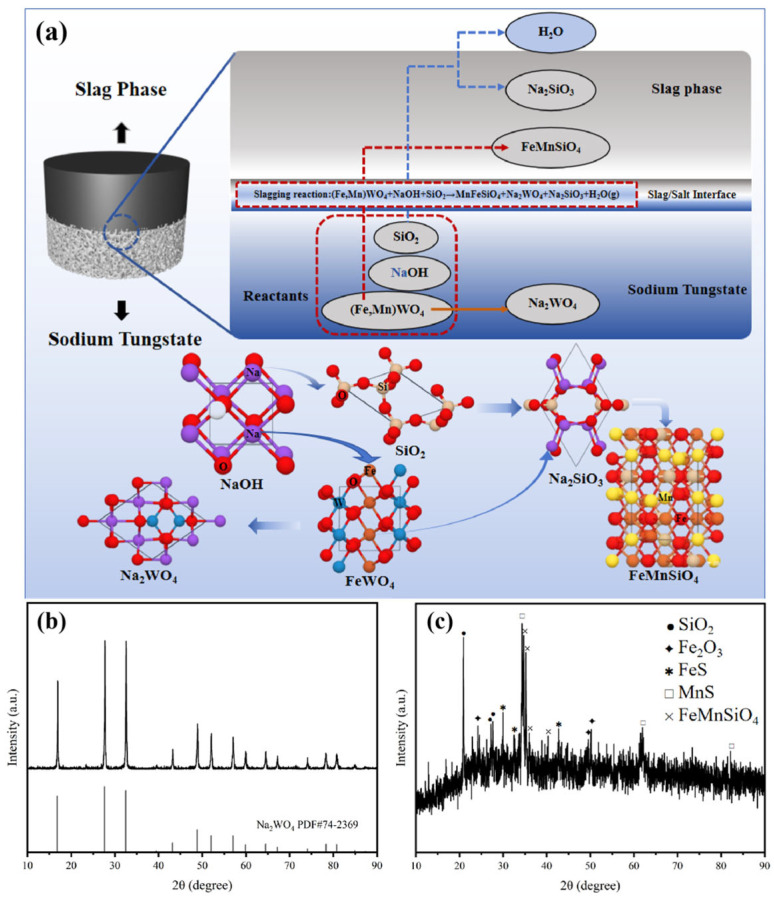
(**a**) Schematic diagram of element migration; (**b**) XRD pattern of the separated salt under experimental condition 4; and (**c**) XRD pattern of the slag phase under experimental condition 4.

This study successfully realized the efficient separation of slag–salt two phases in the process of molten salt treatment of wolframite by adding SiO_2_, which provided a basis for exploring the migration behavior of impurity elements. The two phases after separation were analyzed by XRD phase analysis, and the results are shown in Figure 9. The XRD spectrum of the white salt phase is shown in Figure 9b, which shows that it is a pure Na_2_WO_4_ phase, and the slag phase is shown in Figure 9c: it is composed of iron, manganese, silicon oxides and related composite compounds. The main element trend diagram is shown in Figure 9a, which indicates that W and Na elements in wolframite selectively migrate and enrich in the sodium tungstate salt phase in the lower layer, while the main impurity elements Fe, Mn, Si, S, Pb and Ca are significantly enriched in the upper layer slag phase.

To deeply understand the formation and phase separation behavior of the slag phase in this study, this paper analyzes the experimental results in combination with the isothermal phase diagram of the SiO_2_-Fe_2_O_3_-MnO ternary system at 1000 °C. Under the optimal experimental conditions (wolframite:NaOH:SiO_2_ = 0.0261:0.15:0.05 mol), the Fe and Mn oxides produced by the decomposition of wolframite and the added SiO_2_ together constitute the main components of the slag phase. Ignoring other trace impurities and the fluxing effect of Na_2_O for a preliminary estimate, it can be known from Figure 4b that the composition point of this slag phase is roughly located near 50 mol% SiO_2_, 33 mol% Fe_2_O_3_, 17 mol% MnO. Phase diagram analysis indicates that the experimental point of Experiment 4 falls into the multiphase coexistence region of “Rhodonite + Slag-liq + Spinel”. The thermodynamic characteristics of this region determine that phase separation will occur in the system, forming immiscible silicate-rich melt and oxide phases, which theoretically and effectively explain the slag–salt stratification observed in the experiment. The precipitated stable crystal phases, such as iron-manganese olivine ((Fe,Mn)_2_SiO_4_) and rhodonite ((Fe,Mn)SiO_3_), can serve as “impurity trapping agents”, effectively fixing elements such as Fe, Mn, Si, Pb, etc., in the slag phase.

The key to the successful construction of this system lies in the synergistic effect of NaOH and SiO_2_. Excessive NaOH is not only used for the decomposition of wolframite but also reacts with SiO_2_ to produce sodium silicate (Na_2_SiO_3_). The introduction of Na_2_SiO_3_ significantly reduces the melting point and viscosity of the slag system, making it highly fluid even at a relatively low temperature of 1000 °C, thus promoting the spontaneous separation of slag and salt based on density differences. At the same time, the presence of Na_2_O alters the interfacial characteristics of the slag phase, reducing interfacial adhesion between the slag and molten Na_2_WO_4_, thereby forming a sharp interface that allows the two phases to be readily separated. In summary, the optimal ratio obtained in the experiment is highly consistent with the good phase separation area indicated in the phase diagram. This study, by introducing a NaOH-SiO_2_ regulation strategy, successfully constructed a slag system with low viscosity and high-efficiency impurity fixation capability, providing critical technical support and theoretical basis for the realization of one-step preparation of high-purity sodium tungstate. The XRD results further indicate that the FeS in the slag phase originates from the sulfidation reaction of iron-containing sulfides (such as FeS_2_) in the raw ore with MnO under oxygen-containing conditions, producing FeS and MnS. At the same time, the added SiO_2_ reacts with NaOH to produce sodium silicate, which then reacts with Fe_2_O_3_ and MnO to form the iron-manganese olivine (FeMnSiO_4_) phase. This silicate melt achieves clean separation from the Na_2_WO_4_ molten salt due to their immiscibility and density differences during the cooling process, ultimately forming a clear two-phase structure, providing an effective means for the efficient extraction of tungsten and impurity separation.

The major impurity elements are immobilized within stable silicate phases. Compared to conventional hydrometallurgical residues, which typically exhibit reactivity and solubility, this significantly reduces their environmental hazards.

#### 3.3.3. Product Characterization

**Figure 10:**
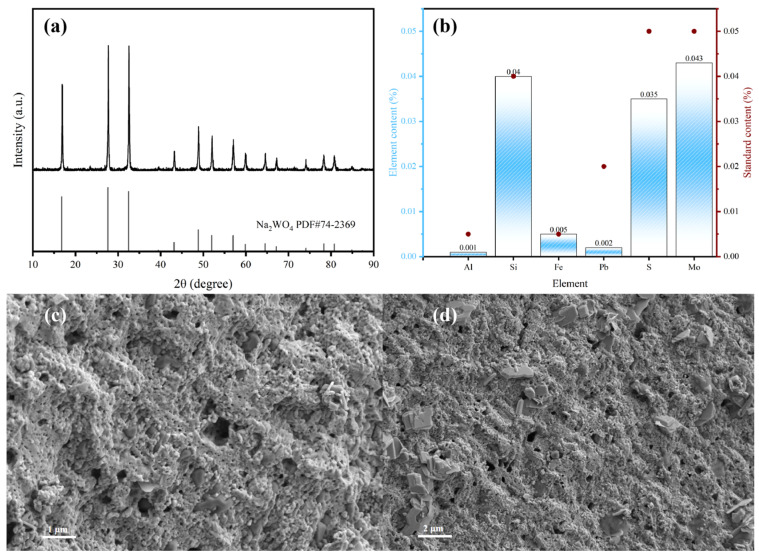
(**a**) XRD pattern of the separated product. (**b**) Comparison ICP diagram of the separated product with the national standard (**c**,**d**) SEM image of that product.

Introducing SiO_2_ into the system under high temperature conditions of 1000 °C can effectively separate the molten slag–salt two phases. XRD phase analysis of the separated products showed that the white salt phase obtained from Figure 10a is pure Na_2_WO_4_ crystal. As can be seen from Figure 10c,d, the product presents a developed porous network structure composed of irregularly agglomerated particles, with clear layered crystals embedded in it. The water generated in the system quickly escapes at high temperatures, forming this porous structure. Further, ICP-OES was used to analyze the chemical composition of the prepared Na_2_WO_4_, and the results are shown in Figure 10b. The Na_2_WO_4_ product has extremely high purity, calculated to be 98.76%, and the content of typical impurity elements such as Al, Si, S, Fe, Pb,Mo, etc., are all lower than the limit standard of market first-class sodium tungstate. The above results show that this method can directly prepare high-purity sodium tungstate through a one-step reaction and has good application potential.

**Figure 11:**
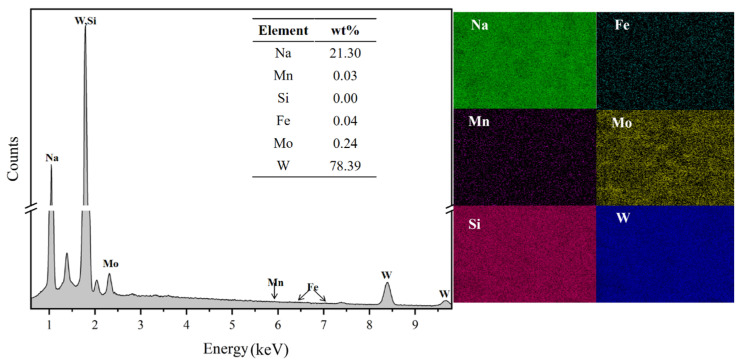
EDS analysis of the obtained sodium tungstate product.

The products were subjected to scanning electron microscopy-energy dispersive X-ray spectroscopy (SEM-EDS) analysis, with results presented in Figure 11. Elemental mapping revealed a uniform distribution of Na, W, and O, corresponding to the Na_2_WO_4_ phase. Notably, signals from major impurity elements Fe, Mn, and Si within the Na_2_WO_4_ particles were either negligible or at background levels. Although EDS provides semi-quantitative data, this observation visually confirms the effective exclusion of these impurities from the salt phase at the microscopic scale. This finding is consistent with ICP-OES quantitative analysis results, collectively providing strong evidence for the directed migration of impurities into the slag phase during the separation process.

### 3.4. Summary and Evaluation of Process Advantages

Based on the results from Section 3.3.1, Section 3.3.2 and Section 3.3.3, it can be concluded that this process demonstrates significant advantages over traditional wet metallurgical methods in wolframite processing. Environmentally, by eliminating aqueous medium processing, this technology effectively avoids generation of high-salinity wastewater along with associated treatment costs and risks. Hazardous impurities are converted into stable silicate phases, while the resulting slag phase can be reutilized as construction materials. Through hazardous waste management at source, the process aligns with principles of green metallurgy.

Operationally, single-step separation significantly simplifies the technological flowsheet. Compared to conventional wet processes, multiple procedural steps are eliminated, thereby reducing both capital and operational expenditures while enhancing process stability.

This study not only provides a technically viable method for preparing high-purity sodium tungstate (Na_2_WO_4_) but also proposes an intrinsically integrated alternative process combining environmental benefits with practical value, establishing a novel pathway for sustainable tungsten extraction from complex resources.

### 3.5. Preliminary Assessment for Scale-Up and Industrial Application

Based on the process advantages demonstrated at laboratory scale, a preliminary assessment of its scaling-up potential has been conducted. In terms of energy consumption, the moderate operating temperature of 1000 °C is significantly lower than that of conventional pyrometallurgical processes (>1100 °C), which suggests potential reduction in specific energy consumption. Economically, although reagent costs (NaOH and SiO_2_) are relatively high, the highly simplified process flow may offset these expenditures through reduced capital and operational expenditures. Technologically, the core phase separation step relies on conventional high-temperature furnace technology, providing a clear pathway for industrial implementation. During scale-up, particular attention should be paid to optimizing reagent ratios for feedstocks with varying compositions, while developing resource utilization strategies for silicate slag to enhance sustainability.

## 4. Conclusions

This study proposes a novel low-temperature alkali fusion phase control method for the efficient and clean extraction of tungsten from wolframite. The core innovation lies in the use of SiO_2_ as a phase regulator to achieve spontaneous slag–salt separation. In the NaOH flux system, the introduction of SiO_2_ as a slag phase modifier enables the decomposition products of wolframite to separate into an immiscible sodium tungstate liquid phase and a silicate-based slag phase at the relatively low temperature of 1000 °C, thereby achieving efficient one-step slag–salt separation.

The optimal process conditions were determined as follows: a molar ratio of WO_3_ to NaOH to SiO_2_ of 0.0261:0.15:0.05, a melting temperature of 1000 °C, and a holding time of 3 h. Under these conditions, clear two-phase separation was achieved, yielding a sodium tungstate product with a purity as high as 98.76%, which complies with the Grade One standards specified in GB/T 10116-2007.

Mechanistic studies indicate that the addition of SiO_2_ promotes the formation of stable silicate phases (such as fayalite and rhodonite), effectively immobilizing impurity elements (Fe, Mn, Si, Pb, S, Mo, etc.) within the slag phase. Phase diagram analysis confirms that the slag composition resides in a multiphase coexistence region favorable for phase separation. The synergistic effect between NaOH and SiO_2_ not only reduces the melting point and viscosity of the slag but also enhances its fluidity, thereby facilitating clean separation from molten Na_2_WO_4_.

Consequently, this process offers distinct environmental and practical advantages over conventional hydrometallurgical routes. Environmentally, it completely avoids the generation of high-salinity wastewater and transforms hazardous impurities into a chemically stable, inert silicate slag. Practically, the one-step separation significantly simplifies the process flowsheet by eliminating multiple unit operations.

This process achieves efficient extraction while significantly enhancing the environmental sustainability of the metallurgical process. The silicate slag phase formed through SiO_2_ regulation not only effectively enriches impurity elements, but its stable mineral structures (such as fayalite and rhodonite) also endow the slag phase with excellent chemical stability.

As a proof-of-concept laboratory study, the process sensitivity to the SiO_2_ dosage and the need for slag valorization are acknowledged as key considerations for future scale-up. Subsequent work should focus on continuous operation, detailed techno-economic analysis, and the development of slag utilization pathways to assess its full industrial potential.

## Figures and Tables

**Table 1:** XRF results for wolframite.

Oxide	WO_3_	Fe_2_O_3_	MnO	SiO_2_	PbO	CaO	Al_2_O_3_	Bi_2_O_3_	K_2_O
wt.%	62.050	16.544	7.200	4.526	3.199	1.209	0.884	0.276	0.222
Oxide	CuO	Na_2_O	TiO_2_	ZnO	Nb_2_O_5_	P_2_O_5_	ZrO_2_	MoO_3_	Other
wt.%	0.171	0.151	0.121	0.078	0.052	0.038	0.031	0.021	3.175

**Table 2:** Melting Partitioning Ratio Table.

No.	(Fe,Mn)WO_4_	NaOH	SiO_2_
1	0.0261 mol	0.15 mol	0.0222 mol
2	0.0261 mol	0.15 mol	0.0333 mol
3	0.0261 mol	0.15 mol	0.0417 mol
4	0.0261 mol	0.15 mol	0.05 mol
5	0.0261 mol	0.15 mol	0.0583 mol
6	0.0261 mol	0.15 mol	0.0667 mol
7	0.0261 mol	0.15 mol	0.075 mol
8	0.0261 mol	0.15 mol	0.0833 mol
9	0.0261 mol	0.15 mol	0.0917 mol

## Data Availability

The original contributions presented in this study are included in the article. Further inquiries can be directed to the corresponding author.
